# The Vaginal Microbiome of Mares on the Post-Foaling Day Under Field Conditions

**DOI:** 10.3390/ani14223337

**Published:** 2024-11-20

**Authors:** Katarzyna Płoneczka-Janeczko, Marcin Magdziarz, Marta Siemieniuch-Tartanus

**Affiliations:** 1 Department of Epizootiology with Clinic for Birds and Exotic Animals, Faculty of Veterinary Medicine, Wrocław University of Environmental and Life Sciences, Plac Grunwaldzki 45, 50-375 Wrocław, Poland; katarzyna.ploneczka-janeczko@upwr.edu.pl; 2 Hugo Steinhaus Center, Faculty of Pure and Applied Mathematics, Wrocław University of Science and Technology, Wyspiańskiego 27, 50-370 Wrocław, Poland; marcin.magdziarz@pwr.edu.pl; 3 Department of Large Animals Diseases with the Clinic, Institute of Veterinary Medicine, Warsaw University of Life Sciences, Nowoursynowska 100, 02-787 Warszawa, Poland

**Keywords:** mare, microbiota, vagina, perinatal period, next-generation sequencing

## Abstract

The vaginal bacteria are critical for neonatal immunity, as well as for further infections and pathologies in foals and mares during the postpartum period. The vaginal microbiota was examined in six mares. Swabs were taken from the vaginal caudal wall within 12 h after natural delivery. Next-generation sequencing was used to analyze the composition of the vaginal microbiota and identify shared bacterial taxa. The post-delivery vaginal microbiome of mares included 18 identified families, 25 genera, and 33 species. The core families of the mares’ microbiome comprised *Leptotrichiaceae* (21.72%), *Peptostreptococcaceae*/*Tisserellales* (15.54%), *Corynebacteriaceae* (13.32%), *Aerococcaceae* (10.84%), and *Campylobacteraceae* (4.26%). Shared bacterial species included *Corynebacterium kutsheri*, *Campylobacter* spp., *Facklamia* spp., and *Oceanivirga* uncultured bacteria. We found the presence of various *Corynebacterium* and *Streptococcus* species that could be involved in uterine disorders. Further research should focus on the possible pathogenicity of these bacteria for mares in the postpartum period and newborn foals.

## Simple Summary

The present study aimed to detail the vaginal microbiome of mares on the post-delivery day under field conditions using the next-generation sequencing technique (NGS). This method allows for the determination of bacterial species composition at multiple levels and enables the assessment of biological diversity in the examined environment. It is believed that greater bacterial diversity is more beneficial for the physiology of the organ. The bacteria colonizing the vagina may include both saprophytic and potentially pathogenic microorganisms. Bacteria inhabiting the mucous membranes engage in constant dialogue with the host’s immune system and are essential for maintaining balance on the mucous membranes. In the present study, various species of *Corynebacterium* spp. and *Streptococcus* spp. were identified, and their potential pathogenicity to the endometrium should be considered.

## 1. Introduction

The uterine microenvironment has long been considered germ-free, although the likely existence of commensal microbiota within the uterine compartment was assumed. In recent years, thanks to the use of next-generation sequencing techniques (NGS), it has become obvious that the endometrium and placenta host distinct microbiota [1,2]. Some authors dispute the possibility of viable bacterial communities inhabiting the placenta and suggest that the detected molecular signals of bacteria in the placenta are background DNA contaminants or they may reflect bacterial products present in the maternal blood [3,4]. The nature of the colonization of the uterus, cervical canal, and oviduct remains even less precisely explored, especially in species other than humans. Besides a few studies concerning the microbiome of the equine reproductive tract, there is still scarce information regarding equine uterine microbiota [5,6]. The taxonomic representation of the uterine microbiome was found to be similar to that found at the external cervical ostium [7], although samples taken from the placenta have indicated that this microbiome was significantly different from the microbiome found in the vagina, mouth, and feces [6]. The bacterial flora of the vagina in mares is very rich, as proven by ordinary bacterial culture [8,9] or identified with MALDI-TOF [10]. A large number of bacteria colonizing the vagina may be due to its location near the anus, and, therefore, possible contamination of this area with feces. During mating, bacteria located on the stallion’s penis enter the vagina, and bacteria located on the mare’s external genital organs may also be introduced. Most uterine infections develop as a result of the transfer of pathogenic microflora through the vagina, so the composition of the microbiome of this organ is crucial. Previous studies indicate that the vagina may be a source of bacteria that reach the placenta, amniotic fluid, and infant through the choriodecidual plate in humans [11,12]. However, vaginal bacteria are critical for neonatal immunity, as demonstrated by comparisons between caesarean- and vaginal-delivery offspring, highlighting the key role of vaginal microbiota in immune education in infants [13,14,15]. We chose a period of up to 12 h after natural birth, without human manipulation, to examine the microbiome because it may be crucial for the foal’s health during this time. It is well known that, due to the tight placental barrier, antibodies do not enter the blood of the fetus in horses [16]; therefore, the newborn foal is susceptible to so-called environmental infections. Only the proper intake of colostrum rich in antibodies by the foal largely guarantees its resistance to bacteria found in the environment [17]. For this reason, the presence of pathogenic bacteria in the mare’s reproductive tract, which can be transferred to the foal during delivery, is significant for the foal’s development.

Another important issue is the possibility of transmitting pathogenic bacteria ascending into the uterine lumen and causing postpartum metritis, which is quite common in cows [18]. In mares, uterine involution **occurs** much more quickly compared to that observed in other species of domestic animals; however, infection of the uterus during the perinatal period can negatively affect the mare’s ability to conceive.

The aim of our research was to investigate and characterize the composition of the vaginal microbiome of mares up to 12 h post-delivery under field conditions through the sequencing of 16S rRNA in order to determine the core microbiome and the presence of potentially pathogenic microorganisms.

## 2. Materials and Methods

### 2.1. Animals

The procedures conducted in these studies are routine veterinary activities and do not require the consent of the Institutional Animal Care and Use Committee, which was confirmed by the Local Ethical Committee at the University of Warmia and Mazury in Olsztyn with decision #LKE.31.01.2020.

The research was conducted on six mares of Konik Polski Horses, born and raised at a Stud Farm (the Research Station of the Institute of Animal Reproduction and Food Research, Polish Academy of Sciences) in Popielno (53°45′16.4″ N, 21°37′42.1″ E). All mares were mated naturally to the same stallion, following an ultrasound examination. The mares were aged 5 to 16 years and weighed between 360 and 430 kg. The reproductive history of the mares showed no pathologies in the past. The mare’s reproductive organs displayed no abnormalities before mating, and the ultrasound images revealed no visible fluid or other signs of inflammation. The mares gave birth in stalls under supervision, but without human intervention, in the evening or late evening. The first postpartum check-up took place two hours after delivery and aimed to determine whether the placenta had separated, the foal had stood up and collected colostrum, and whether it had expelled meconium. A complete clinical examination, including main vitals (pulse, temperature, and number of breaths), was performed the next morning, approximately 12 h after delivery, to rule out any disease factors. Before a routine rectal examination of the mare’s reproductive organs was conducted, a swab was taken after dilating the labia with a gloved hand. Five of the six mares delivered in mid-March and one on the 15th of April. The inclusion criteria for mares were as follows: use of the same stallion for mating, no signs of inflammation before mating in the ultrasound examination, and no general diseases. The exclusion criteria for the mares used in this study were as follows: a history of retained placenta and clinical signs of endometritis before mating.

The mares were kept in a traditional stable system. During winter and spring, each mare was housed in a single box and released to pasture in a group for 8 h per day. All mares were fed with 8–10 kg of mixed grass–hay/horse/day while in the stable. The hay used for the horses was sourced from cultivable meadows and pastures. The botanical analysis of the forage, as well as the chemical analysis of the hay, has been presented elsewhere [19]. When the horses were in the pasture, they had ad libitum access to grass, water, and mineral licks. When housed in the boxes, they also consumed straw from the bedding material and drank water twice daily in the stable. Licks containing NaCl 94%, Mg 2000 mg/kg, Co 18 mg/kg, Zn 810 mg/kg, Mn 830 mg/kg, I 100 mg/kg, and Se 10 mg/kg were provided to the horses throughout the year.

### 2.2. Sampling

Before a routine rectal examination of the mare’s reproductive organs was performed, a swab was taken after dilating the labia with a gloved hand without prior perineal cleaning. The samples were taken with a sterile swab dipped in sterile 0.9% saline (Sarsted, Copan, Brescia, Italy) [20] after opening the labia with a gloved hand. Swabs from each mare were collected once, in duplicate, by rubbing the vaginal wall (caudal vagina) for 30 s with the swab. A total of 12 swabs (2 per animal) were collected during sampling. The specimens were quickly placed in cryogenic tubes at a temperature of −20 °C for freezing and storage. After the collection of vaginal swabs was completed, the samples were shipped in a styrofoam box with a cooler (at a temperature of 2–4 °C) by a courier service directly for analysis (Genomed, Warsaw, Poland). The swabs were not refrozen.

### 2.3. DNA Extraction

Genomic DNA was isolated immediately after sample delivery using Genomic Mini AX Bacteria (A&A Biotechnology, Gdansk, Poland), according to the manufacturer’s instructions, with an additional mechanical lysis of each sample facilitated by zircon balls in a FastPrep^®^ homogenizer (MP Biomedicals, Santa Ana, CA, Poland), following a previously reported procedure [21]. The concentration of DNA was measured using the fluorometric method with a Qubit 4 fluorometer (Thermo Fisher Scientific, Gdynia, Poland). The presence of bacterial DNA was confirmed with the qPCR reaction, using universal primers 1055F (5′-ATGGCTGTCGTCAGCT-3′) and 1392R (5′-ACGGGCGGTGTGTAC-3′) for 16S rRNA [22]. Demineralized water was used as a negative control. The metagenomic analysis of *Bacteria* and *Archaea* was based on the hypervariable region V3-V4 (encompassing approximately 469 bp) of the amplification of the 16S rRNA gene. For the amplification of the selected region and the preparation of DNA libraries, a pair of primers (341F and 785R), along with NEBNext Q5 Hot Start High-Fidelity DNA Polymerase (NEB) (New England, Biolabs, Ipswich, MA, USA), were used. For the measurement of DNA concentration, 1 µL of a reaction mixture was taken immediately after the PCR. The unpurified product contained the reaction mixture with primers/dimer primers; therefore, the result for the negative control was not zero. A concentration of up to a value of about 1.5 ng/µL was considered acceptable. When the negative control was above this value (indicative of contamination of PCR reagents), the reaction was repeated. Arbitrarily, it was assumed that the lowest DNA concentration for the testing sample should be twice that of the minimal concentration of the negative control. Finally, purification of the samples was performed with AMPure XP (Fisher Scientific, Gdynia, Poland), followed by final DNA measurement using the fluorometric method on the Tecan reader. PCR was conducted to index DNA in 50 µL reaction volumes. Next-generation sequencing was carried out on the MiSeq sequencer (Illumina) using paired-end technology (PE300) by Genomed (Warsaw, Poland). MiSeq Reporter (MSR) software v.2.6 was utilized for data analysis. To ensure the classification of reads at the species level, bioinformatic analysis was conducted using QIIME 2 software, a semiquantitative approach to microbial ecology based on the database of reference sequences SILVA v.138. Data analysis from the NGS was performed, including protocols with all sequences obtained after filtering. The diversity of the vaginal microbiota was analyzed using alpha (Shannon and Simpson) and beta (Bray–Curtis) diversity indices [23]. The diversity indices were calculated for all six mares. The Wilcoxon signed-rank test was applied to compare the microbiota of every two mares. The calculations were performed separately at the family, genus, and species taxonomic levels. All calculations were performed in the MATLAB R2020a environment. Taxa present in amounts equal to or greater than 1% of the total identified DNA sequences in at least one of the individuals were classified as ‘abundant,’ as proposed by Kim et al. [24]. If the proportion of identified sequences was <1%, taxa were classified as ‘nonabundant’.

### 2.4. Alpha Diversity

The diversity of the microbiota in mares was analyzed using two standard alpha diversity indices (Shannon and Simpson) [23].

The Shannon diversity index is the standard statistical measure of diversity within a given community. It is defined as follows:

(1) $$D_{Sh}=-\sum_{i=1}^{R}\;p_{i}\;ln\;p_{i}\;.\;$$

In the above formula, *R* is the richness of the community, i.e., the total number of types in the community. Moreover, *p_i_* is the relative abundance of the *i*-th type, i.e., the proportion of individuals of the *i*-th type found divided by the total number of individuals observed. Note that some authors define *D_Sh_* as the exponent of (1).

The Simpson diversity index is defined as follows:

(2) $${\;D}_{Si}=\frac{1}{\sum_{i=1}^{R}\;{p_{i}}^{2}}\;.\;$$

Here, the notation is the same as that in Formula (1). $D_{Si}$ measures the degree of concentration of the community. The calculations were performed in the MATLAB R2022b environment.

### 2.5. Beta Diversity

Beta diversity was analyzed using the Bray–Curtis index, which is the standard and well-established tool for comparing microbiota. The Bray–Curtis distance between two communities, *A* and *B*, is defined as follows:

(3) $$BC=1-\frac{2C_{AB}}{S_{A}+S_{B}}\;.\;$$

Here, *S_A_* is the total number of specimens counted in community A, *S_B_* is the total number of specimens counted in community *B*, and *C_AB_* is the sum of only the lesser counts for each species found in both communities.

Principal Component Analysis (PCA) was applied to the Bray–Curtis distances in order to detect subgroups of mares with similar characteristics of their microbiota. The calculations were performed in the MATLAB R2022b environment.

### 2.6. Statistical Analysis

Since the analyzed data were not Gaussian, the Wilcoxon signed-rank test was applied, and the corresponding *p*-value was calculated to compare the microbiota of each pair of mares.

## 3. Results

### 3.1. DNA Sequence Data

A total of 5041 evaluated qualitatively operational taxonomic units (OTU) generated using the Illumina MiSeq platform (16S rDNA) were obtained for the mares. The mean OTU per sample for mares was 81,490, ranging from 45,116 to 115,563.

### 3.2. Alpha Diversity Analysis

#### 3.2.1. Species Level

**Figure 1:**
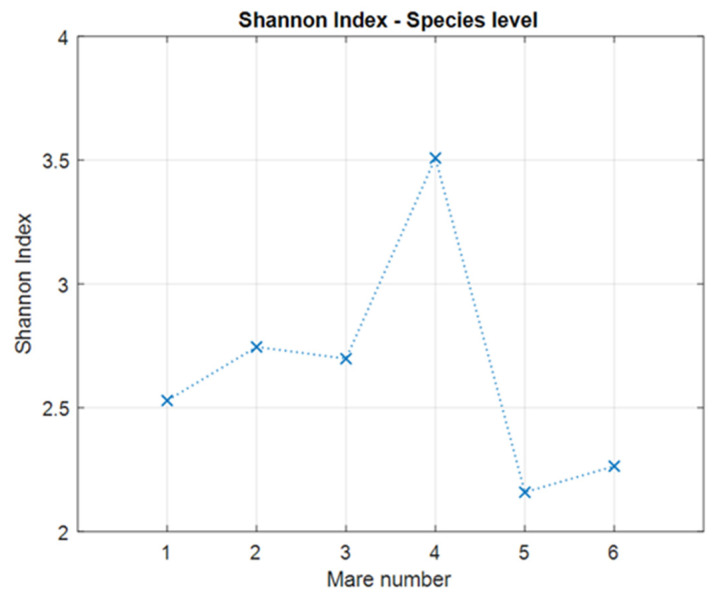
Shannon index calculated for all six mares on the species level.

**Figure 2:**
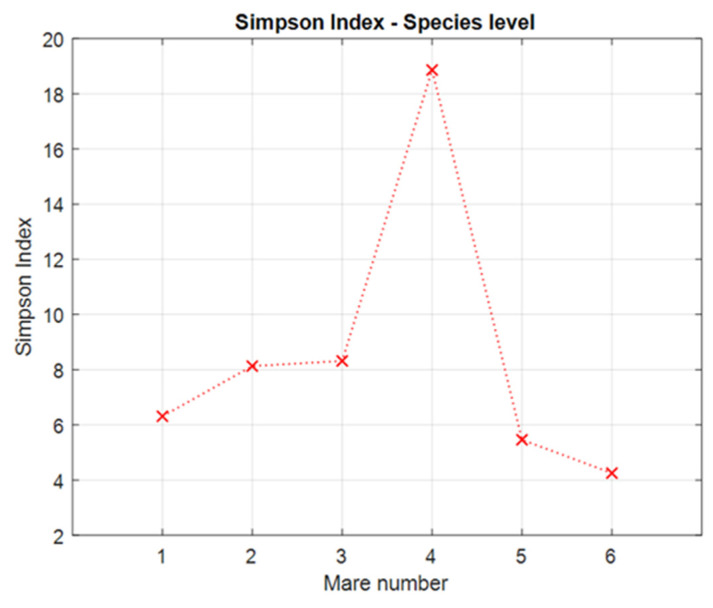
Simpson index calculated for all six mares on the species level.

We observed large differences in the biodiversity of the microbiomes of the six mares. Based on the results obtained for the Shannon and Simpson indices (see Figure 1 and Figure 2), it is noteworthy that by far the highest biodiversity is observed in Mare 4, while the lowest is found in Mares 5 and 6. None of the examined mares have coexisting, apparent diseases, and we did not find or estimate any rational reason why No 4 and 5 create a separate cluster, differing strongly from the other mares.

#### 3.2.2. Age of Mares

We also checked the dependence between the age of the mares and the corresponding alpha diversities. Using the Pearson correlation coefficient, we tested the null hypothesis of no correlation between age and Shannon/Simpson index. In the case of the Shannon index, the *p*-value is *p* = 0.3858. For the Simpson index, *p* = 0.2873. Therefore, we conclude that there is no dependence between the age and the alpha diversity indices.

### 3.3. Beta Diversity Analysis—Species Level

**Figure 3:**
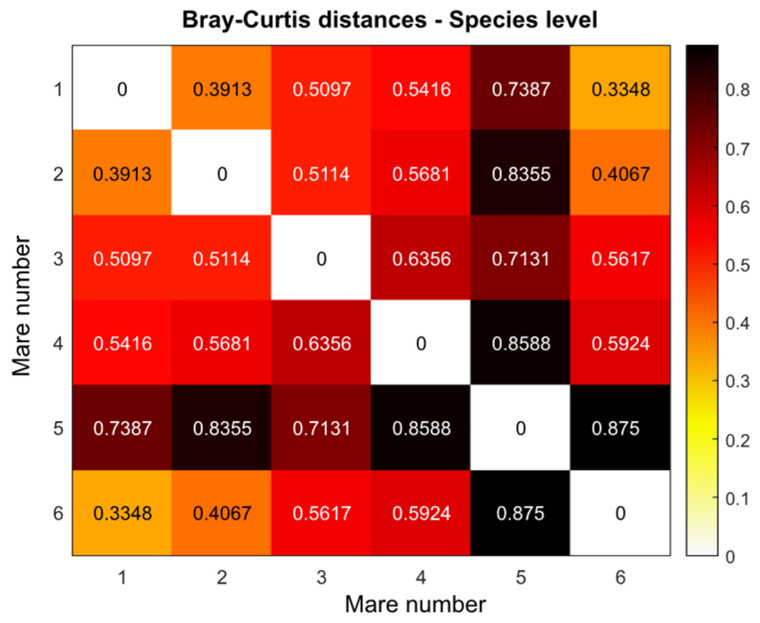
Bray–Curtis distances between all six mares calculated on the species level.

The analysis of Bray–Curtis distances (see Figure 3) also indicates significant differences between the microbiomes of the mares. By far, the largest Bray–Curtis index values (i.e., the greatest differences) are observed between Mares 5 and the others. Additionally, Mare 4 shows a strong difference from the others.

**Figure 4:**
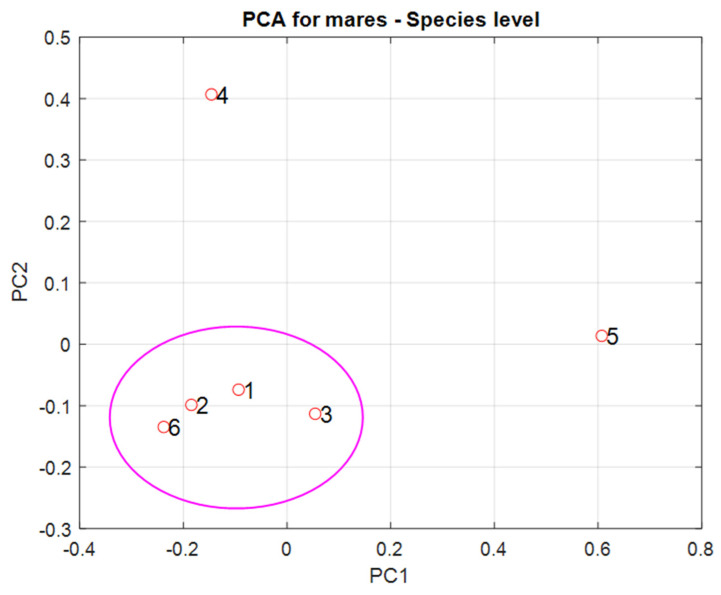
Principal Component Analysis corresponding to Bray–Curtis distances, see Figure 3.

We found confirmation of the above observation in the PCA (see Figure 4). It can be seen that Mares 1, 2, 3, and 6 form a single cluster, while Mares 4 and 5 significantly differ in their microbiome characteristics from the rest.

### 3.4. Statistical Tests

#### Species Level

The paired Wilcoxon signed-rank test was used to compare the microbiomes of each pair of mares. Only taxa with non-zero abundance for at least one individual were analyzed. The *p*-values obtained (see Table 1) confirm that the microbiomes of the mares are significantly different. For all pairs, except two pairs (1–2 and 4–5), the test showed that the microbiome distributions are significantly different from each other. Here, the so-called exponential notation is used, e.g., notation of the form 3.74E−52 means 3.74 *×* 10^−52^.

### 3.5. Microbial Community Composition

The microbial community structures were analyzed for all mares at 12 h post-delivery, including the averages for all animals, as well as individually significant differences. At the kingdom level, the vaginal microbiome predominantly consists of bacteria, for which the average percentage of identified sequences clearly exceeds 90% (97.69%), while the participation of archaea was classified as nonabundant (<1%). Among the identified phyla, the most abundant in the vaginal mucosa were Firmicutes (41.48%), followed by *Fusobacteriota* (25.29%), *Actinobacteriota* (16.80%), *Bacteroidota* (7.69%), and *Campylobacterota* (4.26%).

The most abundant classes inhabiting the vagina were *Fusobacteria* (25.29%), *Clostridia* (22.26%), and *Bacilli* (19.01%), however, *Campylobacteria* were identified in small percentages (4.26%).

**Figure 5:**
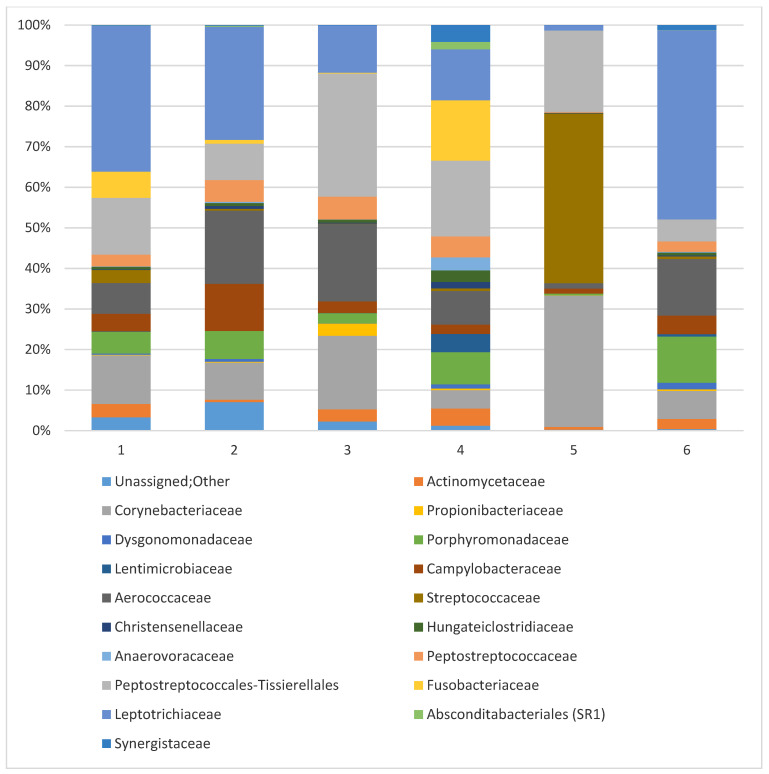
Prevalent families of mares’ vaginal microbiota were identified in individual animals. All sequences assigned to the family taxon are presented, while remaining sequences identified as ‘Nonabundant’ are marked.

At the family level, all mares shared 5 of the 18 identified families, including *Leptotrichiaceae* (21.72%), *Peptostreptococcaceae*/*Tisserellales* (15.54%), *Corynebacteriaceae* (13.32%), *Aerococcaceae* (10.84%), and *Campylobacteraceae* (4.26%) (see Figure 5). Surprisingly, the family *Streptococcaceae* was present as abundant in only two of the six examined mares, varying from 3.14% to 40.43%.

**Figure 6:**
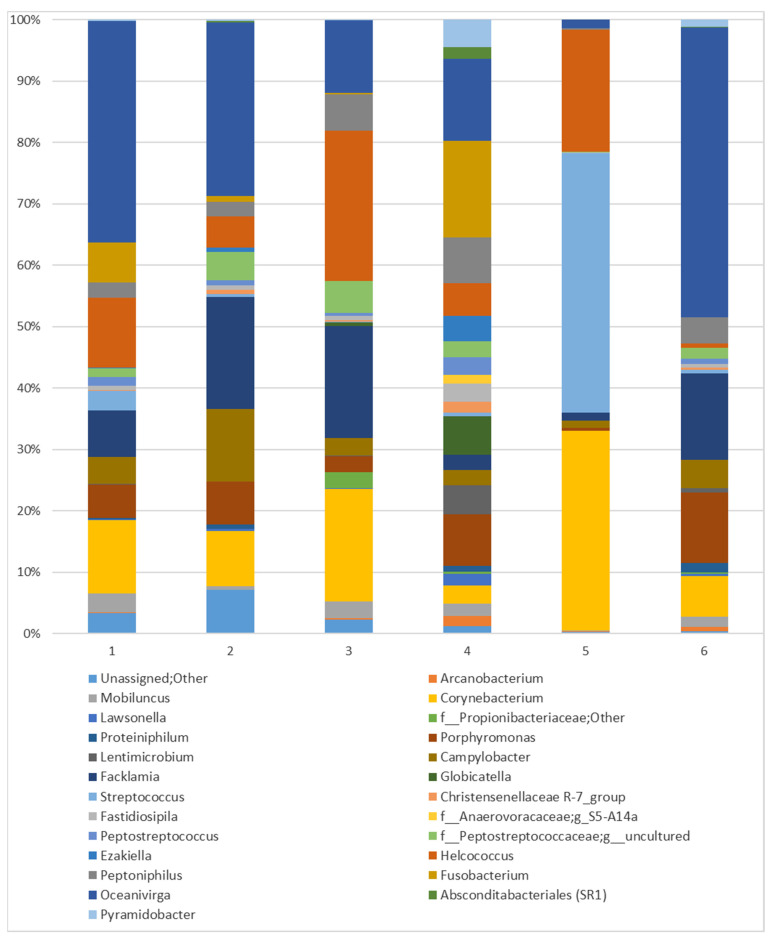
Prevalent genera of mares’ vaginal microbiota were identified in individual animals. All sequences assigned to the genus taxon are presented, while remaining sequences identified as ‘Nonabundant’ are marked.

**Figure 7:**
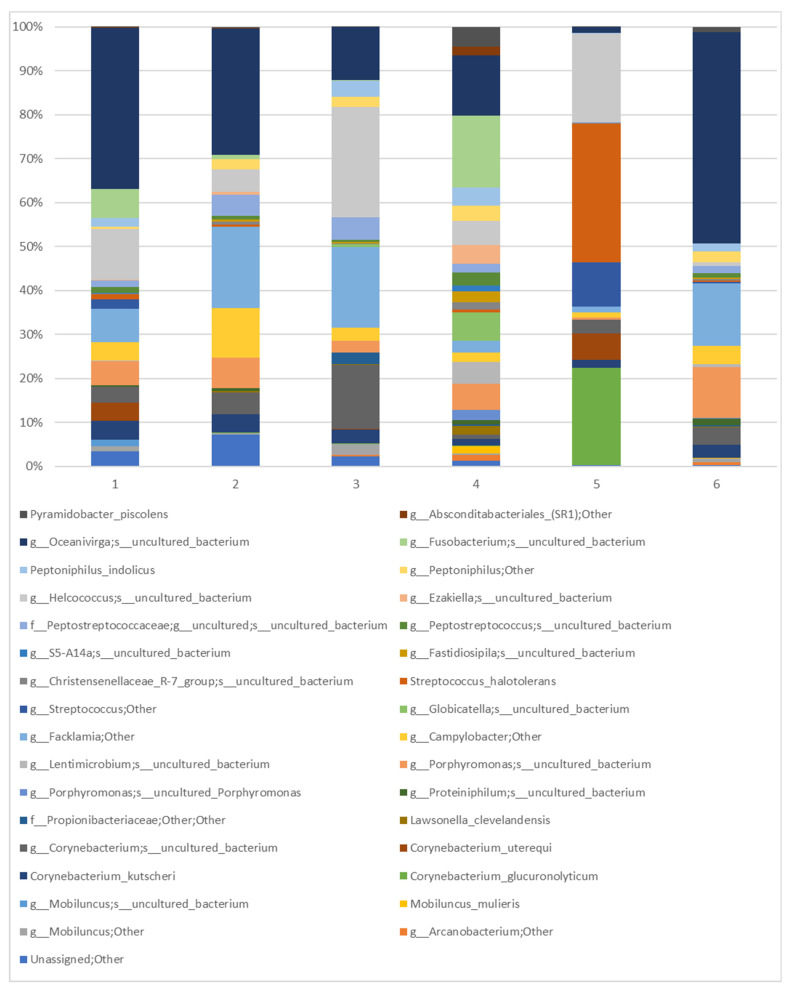
Prevalent species of mares’ vaginal microbiota were identified in individual animals. All sequences assigned to the species taxon are presented, while remaining sequences identified as ‘Nonabundant’ are marked.

The genera representative for all mares include Oceanivirga (21.72%), *Corynebacterium* (12.94%), *Facklamia* (9.73%), and *Campylobacter* (4.26%) (see Figure 6). At the species level, all mares had *Corynebacterium kutsheri* (2.79%), *Campylobacter* spp. (3.95%), *Facklamia* spp. (9.65%), and *Oceanivirga* uncultured bacteria (21.72%) (see Figure 7). It should be noted that the composition of the microbiome in two of the six examined mares is clearly distinguished from the others. Mare No.4 displayed the most diverse microbiome, with 20 identified abundant genera, whereas the range of the same taxa in the remaining four varies from 8 to 12. On the other hand, Mare No.5 demonstrated extremely poor diversity of microbiome, with a high percentage participation of only six genera, while the sequences identified for *Streptococcus* (40.43%) in this mare were the most abundant of all identified in this study.

## 4. Discussion

The mare’s vagina, as expected, was found to be richly inhabited by bacteria. The results obtained through the use of NGS techniques showed that, at the kingdom level, the vaginal microbiome primarily comprises *Bacteria*, while *Archaea* was classified as nonabundant (<1%). The timing of this study is crucial due to the risk of infection passing through the reproductive tract onto the foal during delivery, as well as the potential for developing postpartum metritis, difficulties in uterine involution, and successful conception of a mare during post-foal heat. There has been an increasing interest in characterizing the nature of microbial colonization within the uterus and lower genitourinary tract and its apparent impact on fertility and pregnancy [25]. The mucosal immune system is responsible for interfacing with the outside world, where pathogens can pose a primary challenge. It has been proven that microbiota are involved in the anatomical and functional development of mucosal adaptive immunity [26]. In cows, the predominant bacteria in puerperal metritis, as well as in the normal bovine microflora, were found in the environment. It was believed that the uterine microbiota enriches from the environment when the dam calves [18,27]. It has, however, been shown that the same bacteria that cause metritis are part of the normal bovine bacterial community [18]. Bacteria commonly implicated in equine endometritis include *Escherichia coli*, *Pseudomonas aeruginosa*, *Klebsiella pneumoniae*, *Staphylococcus aureus*, *Streptococcus equi subsp. zooepidemicus*, and other β-haemolytic *Streptococcus* spp. [28,29]. This variety of bacteria, which may be pathogenic, but also includes commensals inhabiting the reproductive tract in mares, causes great diagnostic difficulties. A new player has emerged in horse reproduction, *Corynebacterium uterequi*, which is isolated from the endometrium of mares suffering from clinical and chronic endometritis and experiencing difficulties in becoming pregnant [30] and has been isolated from the discharge of the mare’s genital tract and uterus [31]. Members of the *Corynebacterium* genus are known to exist as commensals, forming part of the normal host microbiota [32]; however, *Corynebacterium* spp. are increasingly recognized as pathogens [33]. Representatives of the *Corynebacterium* genus were isolated from the reproductive tract and placenta of mares in cases of inflammation and abortion [31]. *Corynebacterium* spp. were present in the mare’s vaginal microflora in the current study and constituted the vaginal ‘core.’ Further research is necessary to more precisely determine the pathogenicity of the bacteria in this genus.

In healthy mares, the bacterial culture showed that there are changes in the bacterial microbiota of the mare’s vagina throughout the normal estrous cycle. The dominant bacteria were *Escherichia coli* and *Streptococcus zooepidemicus*. *E. coli* was especially dominant in maiden mares compared to those that had foaled [9]. These results are very significant considering that these bacteria are the main source of infection in equine endometritis [28,34,35].

The core equine vaginal microbiome, examined during the estrous cycle in Arabian mares, consisted of *Firmicutes*, *Bacteroidetes*, *Proteobacteria*, and *Actinobacteria* at the phylum level, which aligns with the results obtained in the mares in the present study. At the genus level, it was characterized by *Porphyromonas*, *Campylobacter*, *Arcanobacterium*, *Corynebacterium*, *Streptococcus*, *Fusobacterium*, *Kiritimatiaellae*, and *Akkermansia* [5]. However, at the genus level, we found some differences, and the dominant genera identified in the vaginal samples were *Oceanivirga*, followed by *Corynebacterium* and *Helcococcus*. *Porphyromonas* and *Campylobacter* described in Arabian mares were less prevalent in our study, as well as *Mobiluncus*, *Globicatella*, *Streptococcus*, and *Peptostreptococcus*, along with uncultured genera from *Peptostreptococcaceae*, *Peptoniphilus*, and *Fusobacterium*. Comparing the results at the genus level regarding the core microbiome, it was found that the genera *Campylobacter* and *Corynebacterium* constituted the core microbiome in all tested individuals. In our own research, the genera *Facklamia* and *Oceanivirga* also formed part of the core microbiome, while, in the study by Barba et al., they constituted less than 85% [5]. We used six individuals in our research, while Barba et al.’s study was carried out on eight mares [5]. Therefore, in the case of our tests, it is impossible to achieve a threshold above 85% if the same bacteria were not found in all tested mares. The genus *Porphyromonas*, which in Barba et al.’s study was the core microbiome in 87.5% of the tested mares [5], was present in 83.33% in our study. It is possible that if our research included a larger number of individuals, the results would be even more similar. Lactic acid bacteria, which dominate human vaginal microbiota [36], do not dominate in equines and were shown to comprise only 0.18% of the taxonomic composition in estrus and 0.37% in diestrus [5]. The vaginal bacterial community reported in Barba et al.’s study [5] shows similarities to the previously described equine distal gastrointestinal tract fecal microbiome [37], which may lead to the conclusion that the colonization of the vaginal tract occurs mainly due to fecal contamination [5]. In another study conducted on pregnant mares, the most abundant phyla, including *Firmicutes*, *Proteobacteria*, and *Bacteroidetes*, were shared among vaginal, placental, oral, and fecal microbiomes [38].

The composition of the vaginal microbiome is also important due to the possibility of developing placentitis during pregnancy through the ascending route. The microorganisms associated with ascending placentitis include *Streptococcus equi subspecies zooepidemicus*, *E. coli*, *Streptococcus equisimilis*, *Klebsiella pneumoniae*, and *Pseudomonas aeruginosa* [29]. In the study by Beckers et al. [6], samples were collected from the oral cavity, vagina, anus, and placenta of five pregnant mares between 96 and 120 days of gestation to examine the core bacterial communities. Alpha diversity was significant, with the body sites being a compounding variable, indicating there was a difference in richness and evenness in the different microbial populations [6].

During pregnancy, the amnion is the innermost extra-embryonic membrane that surrounds the fetus, forming an amniotic sac that contains the amniotic fluid. An amniotic sac usually breaks spontaneously during parturition, when a shoulder of a crowded equine fetus passes through the pelvic canal. For this reason, the question arises of when the mucous membranes of the newborn are colonized by the microbiota and where they originate from. In human medicine, it has been demonstrated that the microbial flora of the maternal vagina affects the flora of the early period in babies delivered via spontaneous vaginal delivery [39,40].

The limitation of microbiome research based on the 16S subunit is that species-related results, analyzed below the genus level, involve margins of error. Therefore, we are cautious in drawing conclusions about which of the identified bacteria are clearly pathogenic and undesirable. Nevertheless, the determination of the vaginal microbiological core, due to both the potential risk of infection in the foal and the possibility of postpartum complications in the form of postpartum metritis, is crucial for protecting the health of the mare and the foal.

The equine vagina harbors a distinct resident microbiome during the perinatal period, characterized by metagenetics, that may settle the foal’s mucosa during parturition or cause postpartum uterine infection. On the other hand, the vagina may contain commensal bacteria that, after colonizing the mucous membranes of the newborn during delivery, will be responsible for maintaining the proper microbiological balance and enhancing the immunity of the host’s mucous membranes.

## 5. Conclusions

An in-depth analysis of the obtained results allows us to conclude that shared microorganisms, which may be included in the early post-foaling core microbiome of mares, represent bacteria from the families *Corynebacteriaceae*, *Campylobacteraceae*, *Aerococcaceae*, *Leptotrichiaceae*, and *Peptostreptococcales-Tissierellales*, all of which were present in all individuals as abundant taxa. However, marked individualization characterizes all taxonomic levels, where the number of identified taxa differed among the mares.

## Figures and Tables

**Table 1:** *p*-values of the paired Wilcoxon test applied to every two mares. Red indicates *p*-values below the significance level α = 0.05.

*p*-Values of Wilcoxon Test	Mare 1	Mare 2	Mare 3	Mare 4	Mare 5	Mare 6
Mare 1	1					
Mare 2	0.216	1				
Mare 3	3.74E−52	8.59E−36	1			
Mare 4	1.88E−23	1.10E−15	5.78E−16	1		
Mare 5	3.22E−15	3.84E−14	1.21E−06	0.204	1	
Mare 6	2.85E−04	0.038	1.12E−50	3.86E−32	7.02E−25	1

## Data Availability

The data will be shared upon reasonable request to the one of the authors (marcin.magdziarz@pwr.edu.pl).
